# The Sociology of Intra-African Pastoralist Migration: The Case of Tanzania

**DOI:** 10.3389/fsoc.2020.518797

**Published:** 2020-11-10

**Authors:** Christopher Simeon Awinia

**Affiliations:** Centre of Economics and Community Economic Development, Open University of Tanzania, Dar es Salaam, Tanzania

**Keywords:** sociology of migration, intra-African migration, pastoralism, social migration, climate change, Tanzania

## Abstract

Intra-African migration in sub-Saharan Africa is increasingly characterized by migration further and further south of the equator. The study focuses on the relationship between pastoralist migration and climate change, followed by loss of control of epidemics, which can lead to losses of large numbers of livestock. While increasing changes in the natural environment have been widely cited in the empirical literature, less attention has been paid to the social context and sociological factors influencing modern-day climate-induced intra-African pastoralist migration. Using Blumer's structural symbolic interaction as an analytic framework, this study examines how social and cultural perceptions influence subjective interpretations that lead to decision points that trigger intra-African pastoralist migration. Using structural symbolic interaction as an analytic framework to study interrelationships between climate-induced stimulus, symbolic interpretation, and migration response, the study identifies that decisions to migrate to other ecosystems were mediated through symbolic signs with assigned meaning and significance communicated through language, signs, symbols, ritual, and religion, which makes up the overall pastoralist cosmology of migration. That these signs and meanings, which are given symbolic meaning, form the basis of African pastoralist sociology of migration. These are the key factors that explain why some pastoralist households decide to migrate to other ecological zones while others remain in traditional pastoralist lands. This study was guided by two hypotheses. The first is that intra-African pastoralist migration is socially defined based on the social and cultural perceptions and meanings attached to interactions with their increasingly changing natural and social circumstances (H1). The second (H2) is that this meaning is defined and modified through an interpretative process in response to changing social and natural circumstances. The sample examined in the study covered seven districts with a high proportion of pastoralists across four eco-climatic zones. A total of 544 pastoralist households were interviewed. In-depth qualitative interviews and observation studies were conducted in three case-study villages. The study concludes that decisions to migrate to other ecosystems are mediated through symbolic signs with assigned meanings and significances communicated through language, signs, symbols, rituals, and religion. Together, these findings contribute to our further understanding of the sociology of recent intra-African migration.

## Introduction

Pastoralist migration is an important aspect of intra-African migration in the context of increasingly changing circumstances in both the natural and social environment. Social factors are important determinants that explain the increasing trend in intra-African pastoralist migration toward migration to destinations increasingly further south of the equator. This is the case because decisions to migrate from one ecosystem to another are mediated through the subjective interpretation of increasing changes in the natural environment caused by climate change, among other factors. Social interpretation is defined and processed through perceptions of the changing natural and social circumstances. A better understanding of the perceptions and subjective interpretations resulting from interactions with both the changing natural circumstances and with peers would represent an important contribution to the sociology of migration in Africa. In the past, researchers have devoted considerable effort to examining the contribution of physical factors such as climate change, lack of water and forage resources, and increasing loss of control over epidemics in existing grazing areas as factors contributing to pastoralist migration south of the equator. In the present study, for example, 73.25% of respondents in the households surveyed said they could not find solutions to their climate change–induced problems^1^. Women accounted for a slightly higher proportion of those who perceived themselves as unable to find solutions to their climate change–induced problems, with 82% of women who participated in the study reporting that they could not find solutions to their climate change–induced problems.

The prevalence of respondents who felt that they were unable to find solutions to their climate change–induced predicaments means that pastoralist households were left to conclude that their only option in response to the economic stagnation arising from the loss of a large number of cattle due to various climate change–induced and related causes. Pastoralists were, however, not passive in their struggle and interaction with climate change and, more specifically, loss of ecological control (Bowring, 1977; Araujo, 2017). Pastoralist households used modern scientific environment management practices such as tree planting, conservation of water sources, and improvements of pasture land to curb new livestock diseases, enforce land rights and boundary demarcation of pastures, and increase access to veterinary services.

Previous research on pastoralist migration in Africa has not paid sufficient attention to the subjective factors that contribute to their migration. These subjective factors are mediated in socially and culturally defined ways in response to changing natural and social circumstances. A relatively small body of research has looked at issues such as the social circumstances, culturally defined interpretations, and personal perceptions that come to bear in response to various types of social stimuli encouraging migration.

This article aims to overcome some of the limitations of the previous work. First, it contributes to our understanding of the social determinants behind the increasing trend for pastoralists to migrate south of the equator in response to changing natural and social circumstances. Second, it seeks to explain how various ways of coping with increasing climate change are culturally perceived and socially interpreted by migrating communities. Third, it seeks to answer why some pastoralists have remained in traditional pastoralist lands while others have migrated. The study takes into account the fact that the decision to migrate is based on subjective interpretations and represents a response to changing circumstances. Interpretations of these changing circumstances vary and, to a large extent, depend on culturally and socially defined perceptions of observed external stimuli. Fourth, the study seeks to contribute to our understanding of the sociology of intra-African pastoralist migration and its place in global migration.

Using the theory of symbolic interactionism proposed by Blumer (1963), the study identified the subjective factors that determine an individual decision to migrate. The study findings illustrate the importance of interpretation: human beings do not respond passively to stimuli but interpret or define them—including each other's actions—instead of merely reacting to them. The study conducts an investigation of the contribution of the following determinants of social migration, as specified by Blumer:

- Human interaction
- Interpretation or definition rather than mere reaction
- Response based on meaning
- Use of symbols
- Interpretation between stimulus and response

Blumer's symbolic interactionism can be schematically expressed as follows:


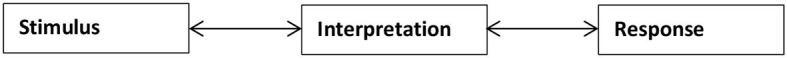


Blumer's unique contribution came from the understanding that human beings interpret or define each other's actions instead of merely reacting to them. He argued that human actions are not merely a response to the actions of others but are, more importantly, determined by the meaning they attach to such actions. He further argued that such human interpretation was guided by the use of symbols in ascribing meaning to the actions of others.

This study draws on Blumer's theory of structural symbolic interactionism to examine first the primary level of human interpretation of changes in the natural world and then the secondary level of interpretation in terms of how meaning is ascribed to these changes, and the collective consciousness of the possibility of migrating develops as a function of the interpretation of these changes. According to Blumer, human consciousness arises from the ability of humans to make meaningful decisions in response to their surroundings, which include both the social and the natural environment.

The findings of the study point to an increase in southward migration among pastoralists in Tanzania and show that the symbolic interpretations of certain climate change–induced characteristics were the main stimuli that triggered the migration response (National Bureau of Statistics Tanzania, 2006).

## Review of the Empirical Literature

According to Western and Manzolillo Nightingale (2003), increasing climate change and extreme weather conditions and variability aggravate the loss of ecological control and give rise to a myriad of livestock diseases. They cite the loss of control over epidemics among livestock as a major push factor prompting pastoralists to migrate to new ecological areas. This interpretation and the sociological meaning informing the decision-point to migrate is myriad in symbolic meaning and signs assigned with the particular characteristics of nature and the way they change between predictable weather conditions and states of climate disequilibrium brought about by increasing climate change and extreme weather conditions. They further found that decisions to migrate were normally reached after people living in formerly predictable climatic states could no longer cope with the new challenges brought by new states of climatic disequilibrium. Individual subjective perceptions of what were predictable weather conditions and what were not were important factors in decisions to migrate (National Bureau of Statistics, 2014).

According to Gifford-Gonzalez (2015), livestock and other herbivore populations compete for forage resources in the rift valley, and their rate of energy intake is reflected in their rates of reproduction and mortality, so their population will fluctuate in climate conditions of disequilibrium compared to a stable state.

Butt (2016) took this argument further by adding that the loss of forage resources and pasture through increasingly deteriorating weather conditions and multiple emerging uses of rangeland also meant that animals increasingly needed to graze on degraded pasture, thus exposing them to the risk of a variety of soil-borne diseases.

This premise was aptly supported by Boone and Wang (2007) who found that climate disturbances increased the prevalence of vector-borne diseases, as they created favorable conditions for vector breeding, the proliferation of pathogens and parasites, and, thus, the transmission of new diseases. Consequently, this affected livestock herd sizes, as survival rates drastically fell during prolonged drought seasons due to diminished immunity.

In their work, Boone and Wang (2007) demonstrate that climate change and extreme weather conditions affected the balance between nature and livestock, as dry months and even dry years meant cattle did not have the prospect of consuming the same amount of forage as in previous years. Their analysis consequently led Boone and Wang to their well-known hypothesis that the lower quantity and quality of forage resources resulting from the effects of climate change lead to less conversion and transformation of the forage into energy within the bodies of cattle and therefore to decreased levels of immunity to disease and lower survival rates (Boone and Wang, 2007). Their analysis shows that livestock herders in East Africa are not passive in the face of the impacts of climate change, but rather perceive and interpret them in their own way. These perceptions and interpretations inform their decision to migrate, as shown in Table 1.

**Table 1:** Perception of climate change impacts among pastoralists and livestock herders.

**Level of impact**	**Mean scores**
**Perception of temperature variability**
Hot dry season temperature has increased	4.7
Main rainy season temperature has increased	4.1
Temperature has increased during dry season	4
Cold dry season temperature has increased	3.6
Small rainy season temperature has increased	3.1
**Perceptions of rainfall variability**
The onset of rainfall has become more unpredictable	4.7
The cessation of rainfall has become more unpredictable	4.5
Drought occurrence frequency has increased	4.5
Rainfall amount has decreased	4.4
Number of main rainy season days has decreased	4.2
Number of small rainy season days has decreased	2.6

*Source: Boone and Wang (2007)*.

It can be seen that livestock keepers' perceptions of their experience of climate change vary by season, particularly with respect to rainfall and rising temperatures, both of which emerge at the top of the respective lists.

According to Blumer (1963), these perceptions do not only arise from human interactions with nature. They are also informed by social interactions with peers, which are decisive in determining the collective response to such increasing changes to their natural and social circumstances. The levels of impacts identified by Boone and Wang (2007) may be in the natural environment, but their interpretation is social and cultural. The types of impacts are defined and given symbolic meanings as signs of failed livelihood systems and indicators that the time has come to migrate. Symbolic significance is ascribed to these signs through culture, interactions with peers, language, and religion. Ogalleh et al. (2012) support this view by asserting that indigenous people usually report their perceptions of climate variability in light of observed impacts to their natural circumstances. People's perceptions are everything to them and play an important part to changes in their social circumstances through making interpretative risk assessment and the decision to migrate.

Boone and Wang (2007) report that droughts, rising temperatures, and reductions in the volume of rainfall were the main perceived impacts of climate change among pastoralists and cattle herders. Other impacts were the unpredictability of both the onset and cessation of the rainy season. Perceived non-climate stressors included increased animal and human population pressure, rangeland deterioration, and weakening of traditional rangeland management. The depletion of water and pasture resources in the area are key factors that pastoralists take into account when deciding to migrate (Butt, 2016). These factors, including perceptions regarding the rising frequency of occurrence of droughts, contribute to decisions to migrate to other ecological areas in search of “greener pastures” in the literal meaning of the expression (Oba, 2009; Desalegn et al., 2017).

Tafesse and Samson (2009) report that coenurosis is a newly emerged disease prevalent in the dry seasons that affects the brains of goats and cows. In addition to coenurosis, PPR (*peste des petits ruminants*), CBPP (contagious bovine pleuropneumonia), tick infections, FMD (fibromuscular dysplasia), trypanosomiasis, salmonellosis, liver diseases, tumors, bleeding and bloating, sudden death, blackleg, and anthrax have also been reported to be sensitive to climate change and as being on the increase. Consequently, these diseases contribute to the loss of control of epidemics, causing mass livestock deaths.

Fratkin (2001), Oba (2012), and Mercandalli and Losch (2017) suggest that, cumulatively, these factors contribute to increased stress on pastoralism as a livelihood system and can lead to decision points at which pastoralists choose to migrate to new, untested ecological zones. Their analysis argues that the loss of forage resources results in low energy intake by livestock, which also affects the capacity of livestock to reproduce and develop immunity to diseases. In addition, livestock lose the capacity to travel long distances. Other effects of loss of ecological control cited by pastoralist households were loss of cattle from dehydration due to a lack of water. This was especially the case during the dry season when it is not possible to rely on transhumance as a traditional coping or adaptive mechanism for extreme climate variations. Moreover, multiple land-use patterns have blocked traditional transhumance corridors (Ståhl, 2015).

Coppolillo (2000) observed that migration among Sukuma agro-pastoralists in Tanzania contributed to a decrease in livestock productivity in terms of milk production. This was because of the lack of availability of water, especially in the dry season, and the increased migration distances traveled by herds in search of forage resources and water. The effects of climate change and micro-migration, which involve traveling further from home, often in large herds and across corridors of high settlement density, affect intake rates, foraging behaviors, milk yields, and livestock body conditions:

Cattle from larger herds were observed to walk more while actively foraging, taking more than ten steps without taking a bite. The increased walking of large herds explains why they range further from home and its effect on intake rates and milk yields (Coppolillo, 2000, p. 91).

According to Bank and Fröhlich (2018), as pastoralists continued to migrate in the midst of confrontation and crisis, pastoralist migration in Tanzania was quickly becoming an international issue. The International Organization for Migration (2018) documented the fact that groups of pastoralists from Tanzania had already been observed in neighboring Zambia and Mozambique, causing conflicts with alternative land users on the way. These conflicts inevitably had profound and lethal dimensions compared with those within Tanzania (Choe, 2007; Bommes et al., 2014).

Fratkin (2001) identified the following as contributing factors to increasing the out-migration of pastoralists from their traditional lands: human and livestock population growth, loss of herding land by pastoralists, increases in the size of land being allocated to protected, gazette, and wildlife management areas as well as hunting blocks and game parks, and urban growth, compounded by drought, famine, and land-use conflict between farmers and those using the land for other purposes.

Awinia (2015) states that the main dilemma faced by pastoralist households was whether they should decide to face vulnerability in existing areas by trying out various coping mechanisms or embracing mitigation measures, including micro-migration, or if they should migrate to areas completely outside of their present climatic zones in search of pasture resources, water, or improved human and livestock health conditions. Migration has been a popular choice for an increasing number of pastoralists, particularly in the context of increasing number of surviving livestock herds, thanks to improved rural veterinary services (Venugopal et al., 2018).

A report by the World Travel and Tourism Council (2018) observes that the wildlife population has also dramatically increased following increased conservation and management measures, including wildlife epidemic control. The combination of increased livestock and wildlife populations, growing human settlements, agrarian reforms, expanded acreage under cultivation, and climate change has contributed to a state of disequilibrium through increased density of herd size and dwindling forage resources, leading to adjusted pressure on existing grazing lands.

## Data Collection Methods

The study was guided by two hypotheses and used mixed methods to triangulate data sources in order to achieve in-depth understanding of the dynamics of social and cultural perceptions and meanings attached to interactions leading to pastoralist migration in Tanzania. We used the following methods to collect data: secondary and archival data sources, administered semistructured quantitative interviews to 544 households in 7 pastoralist districts, held 17 focus group discussions (FGDs) with pastoralist families, and interviewed 54 key informants (KIs)^2^, ^3^. Quantitative data were supplemented by in-depth, open-ended qualitative data collected from 54 KIIs and 17 FDGs, as shown in Table 2 below^4^.

**Table 2:** Study respondents by type of data collection method and district.

**District**	**Households**	**KIIs**	**FGDs**
Bunda	85	08	04
Geita	69	08	02
Hanang	35	04	02
Iringa (Rural)	86	07	01
Longido	59	09	03
Kiteto	90	10	02
Mvomero	120	08	03
Total	544	54	17

### Ethical Statement

Ethical considerations, as articulated by the Open University of Tanzania (OUT) Research Ethics Policy, were taken into consideration when conducting the study. The study's research objectives were clearly read out to respondents before the interviews were conducted. All study respondents were asked to provide their consent before the interviews^5^.

All published materials and secondary data sources that have been cited are in the public domain; copyright permits citation as long as the sources are duly acknowledged.

## Study Findings

The information obtained from the interviews with pastoralist households showed that there was evidence of increasing interaction between pastoralists in the context of increasing changes to their natural environment and habitat. The more frequent livestock epidemics, deaths, and declining average numbers of livestock owned by households affected their perceptions of the desirability of either staying or migrating to other areas. The death of a large number of livestock contributed to the social fragmentation of the extended pastoralist homesteads known as *bomas*. This fragmentation altered the existing culturally defined traditional social arrangements within the *boma*. Gender relations and age-set systems were a case-in-point, as perceptions of the traditional gender division of labor existing among *bomas* were affected.

The fragmentation of what had previously been extended families was driven by increasing vulnerability to changing natural circumstances, which encouraged micromigration in search of water and forage resources. Micromigration continually changed community perceptions. It encouraged homestead fragmentation, which had previously been regarded as taboo. It changed community perceptions in favor of micromigration, and later long-term or permanent migration. In the process, the deeply held symbolic meanings attached to extended family, gender, and age systems, and traditional lands were transformed. A female respondent informed the study about the changes in perceptions that were taking place.

Since we migrated to the Msowero area, our traditional relations between men and women have changed. Because there are fewer youths in the *boma* as a result of migration, women sometimes have to do some tasks in areas traditionally reserved for men. We now have to herd small livestock such as goats and sheep ourselves. Our culture did not allow us to carry out these activities in the past, but nowadays, the culture is being relaxed due to migration away from our larger clan heads and the new realities where we now live^6^.

The study findings show that pastoralist households experienced changes in gender relations as shown by Table 3 below.

**Table 3:** Extent to which perceptions of gender equality among pastoralist men have changed.

**Extent of changed perceptions of gender equality**	**(%)**
Not at all	10.7
To a small extent	20.11
Average	27.86
To a large extent	22.69
To a very large extent	2.4
Do not know	16.05
No response	0.18
Total	100

It can be seen from Table 3 that there was a gradual shift in favor of gender equality.

The study findings show that the reduced numbers of livestock were one of the changes brought about in the course of the interaction between pastoralists and their changing natural circumstances. Although pastoralists were apt to keep large numbers of livestock (50 or more), stock sizes among households were deteriorating due to the increasing incidence of epidemics associated with climate change. Among the households interviewed, 23.06% and 18.63% of pastoralists reported owning herds numbering 50 cattle or more per household and between 25 and 50, respectively. However, some respondents reported diminished stocks, indicating the impacts of climate change, loss of forage resources, and epidemics on livestock survival.

Declining stock size arising from sudden death due to changing circumstances in the natural environment was not just taken passively. It carries a symbolic significance regarding the level of sustainability of the pastoralists' livelihood. This was aptly put by a pastoralist interviewed for the study:

Nowadays, the sun comes out stronger during the dry season, causing all the grass in Mkurlu village to dry. As a result, our livestock become thin and sick. When the sun is really strong, large numbers of cattle starve and die. In our culture, when a large number of livestock start to die, it is a sign that we must migrate to another area.

It can be seen that the pastoralists look to grass and rainfall for signs. Their interaction with nature is interpreted in terms of culturally and socially defined perceptions for a sign as to whether or not to migrate.

The study findings also confirmed that climate change and migration result in the fragmentation and nuclearization of pastoralist families. There was an increase in small nuclear households, with 18.08% of the pastoralist households interviewed having only five household members or fewer. Community members responding to the qualitative FGDs attributed this phenomenon to increasing climate change and extreme weather conditions.

Extended pastoralist households known as *bomas* are configured to serve a number of functions, including the age-set system and gendered division of labor. In the *bomas*, male youths serve as *moran* warriors to safeguard against predators, theft, and tribal conflicts. Inter-tribal conflicts usually revolve around cattle rustling and retaliation. *Bomas* also served as a way to pool livestock at different locations against risk from epidemics, which could wipe out an entire stock. They also served as a way to pool labor to clear and construct new settlement areas in case of migration. Smaller, nuclear family–based homesteads did not have the advantage of scale to fulfill these functions.

Moreover, high mobility of livestock through pastoralist migration aggravated the spread of cattle diseases such as bovine pleuropneumonia, pasteurellosis, and cattle respiratory complex disease. This risk arises from the increased interaction between livestock owned by pastoralists and animals from different regions, including areas pastured by wild animals. The pastoralist households interviewed for the study attributed the large number of livestock deaths that occurred to multiple factors in the course of their interaction with the natural environment, as shown in Table 4.

**Table 4:** Reasons cited for the cause of large-scale deaths of cattle.

**Reason for loss of large size of cattle**	**Yes (%)**
Intermittent livestock epidemics	45.2
Loss of water resources	38.38
Pasture/forage loss	57.2
Livestock theft	36.16
Conflicts with other pastoralists	24.72
Other	11.25

More than half of pastoralist households (57.2%) said that inadequate pasture and forage resources were a leading cause of the death of a large number of livestock experienced by their households. This attests to their perception that the nature of their interaction with the natural environment had a significant relationship to the loss of a large number of livestock. Table 4 also shows that 45.2 and 38.38% perceived intermittent livestock epidemics and loss of water resources, respectively, as accounting for the loss of a large number of their livestock.

Table 4 also shows the perception that the loss of a large number of cattle also arises from social interactions with peers, with 36.16% reporting the loss of a large number of cattle from theft and 24.72% from conflicts with other pastoralists.

Such interactions contributed to the changing perceptions of the ability of households to respond adequately to their predicaments as shown by Table 5 below.

**Table 5:** Pastoralist household perception of their ability to respond to their predicaments.

**Extent**	**Frq**	**%**
Not at all	65	11.99
To a small extent	108	19.93
To a modest extent	175	32.29
To a large extent	90	16.61
To a very large extent	3	0.55
Do not know	101	18.63
Total	542	100

There is a high level of inertia in terms of responding to their predicaments, with 73.25% of the pastoralist households surveyed reporting that they could not find solutions to the predicaments they faced in the course of changing natural and social circumstances. The information provided by Table 5 shows the majority of respondents shared the perception that they were likely to just succumb to the vagaries of economic stagnation and loss of cattle from various climate change–induced and related causes. In terms of the sociology of migration, it is important to note that the alternative to this inertia was to migrate.

The information provided by Table 5 agrees with the Blumerian explanatory model, in which the individual response to migration does not happen in isolation, with the individual as a passive actor. Instead, the decision to migrate is made through interaction between the individual and the changing natural and social circumstances with his or her peers, the perceived meanings that the individual ascribes to such interactions, and responses based on perceptions attached to changing circumstances. A careful study of such interactions, perceptions, and responses is an essential part of the sociology of migration.

While the majority of the respondents reported under Table 5 shared the perception that they could not respond to their changing natural and social predicaments, there was evidence that pastoralists were not passive in their interaction with changing natural and social circumstances. Various response mechanisms were reported. These included applying modern scientific environment management practices such as tree planting, conservation of water sources and pasture land, and better protection of land rights and boundaries, including the demarcation of pastures. Other mechanisms mentioned included increased access to veterinary services to curb new livestock diseases. The dominant perception was that when these perceived responses to their predicaments failed, the last option was migration.

The findings of the study show that there were strongly held perceptions of the importance of protecting traditional pastoralist lands from being converted to other competing land uses. A pastoralist respondent in one of the study's FGDs who previously lived in Uwiro village said they had gradually lost their land to Meru cultivators because they did not possess titles to their grazing land.

We used to live in the Uwiro area. The place had more grass and water springs than Mkurlu. However, each time when we left to graze in the Hanang or Bomang'ombe areas, we found Meru people had settled in our grazing land while we were gone. Over the years, we have now been displaced to Mkurlu, which is drier.

The study established that when pastoralists could not adapt through transhumance migration, they responded through other measures to adapt and assert ecological control. This was often done with the help of pastoralist non-governmental organizations (NGOs). An innovative response measure adopted by pastoralists in the case-study pastoralist village in the Mkurlu lowland area was the adoption of a rotational grazing system. This adaptation measure involved parceling pasture areas into grazing blocks, monitoring grass growth in the blocks, and grazing cattle by rotation around the blocks. This system contributed to reducing land carrying capacity by restricting grazing in the parcels to allow forage resource growth. This adaptation is a microcosm of the traditional transhumance system. It allowed grass to grow between rotations. As a grass monitoring scout at Mkurlu village put it.

We have been appointed to monitor the grass. We have parceled the land into blocks. We come to physically observe the blocks to determine the thickness of grazing grass. We then announce to the village that all herds should be grazed in a certain block. This allows grass in the other conserved blocks to grow in quantity and quality. Before this system was put in place, you would have found the grass-cover had been depleted in all of this area to the extent that you could see the bare soil.

The foregoing shows that the interaction with nature in increasingly changing natural circumstances provides a symbolic meaning to grass in pasture lands. Changing natural circumstances are interpreted in terms of the density of sun rays and the quality and quantity of forage resources. These parameters were signs with symbolic meanings that were significant to the extent that the community appointed scouts to monitor them so as to regulate their interaction with increasingly changing natural circumstances. This meant that the changing natural circumstances also defined changes in the ways the communities interacted with their peers.

Another observed adaptation of transhumance was that, instead of the whole homestead migrating during the dry season, now men were migrating by themselves, leaving women, and children behind in order to safeguard their land from further encroachment by agriculturalists from Uwiro village. However, once these adaptation measures were no longer adequate to respond to the problems encountered in interactions with their natural circumstances, pastoralist households typically started to contemplate migrating to other completely new ecological areas. This becomes the tipping point for migration into other areas where ecological control can be regained and exercised.

The qualitative responses from the open-ended questions in the semistructured household questionnaire indicated that the most frequently cited reason for the loss of a large number of cattle was that they died by falling while climbing either down or up the river valley, on their way down to drink water, or on their way back (11.48%). This indicates that the receding water resources meant that pastoralists had to further away to more remote water sources to access drinking water for livestock. This was particularly the case during the dry season. A pastoralist head of household informed the study during one of the FGDs that:

About one out of every five cattle are at risk of dying or falling ill due to exhaustion, dehydration, and lack of nutrients arising from low forage intake. This is because instead of grazing, they eat grass while walking. Migration, whether to other areas in the vicinity or to far distant lands, has a cost in terms of cattle lost. The further the herds are taken for pasture, the greater the risk of sickness and death.

Other causes of loss of a large number of cattle mentioned by the study respondents were protracted labor (6.56%); eating poisonous grass, including cassava (1.64%); mixing with other cattle herds and thus being exposed to epidemics (3.28%); and being killed by ranch or enclosed wildlife game range owners (1.64%).

The study further found that increasingly changing natural circumstances reduced the average stock size of cattle among households. Although identifying themselves and living primarily as pastoralists, 16.61% of households reported having only 5 or fewer cattle, and 27.31% had 6–15 cattle, which is considered small by pastoralist standards in Tanzania. The study found that this diminishing stock size was one of the main determinants that triggered the pastoralists' decision to migrate to other ecological zones. The loss of a large number of livestock due to epidemics carried a symbolic meaning signaling the immediate need to migrate from the present settlement.

This notion was conveyed by one of the study interviewees, who said

According to our tradition and culture, when a sizable herd of livestock starts dying over a short period of time, then it is time to immediately vacate the area. This is because the deaths could indicate that there are pathogens in the vicinity (environment) that could wipe out the entire herd and also infect humans.

The study found that changing forms in each of these characteristics provided a framework within which the decisions that triggered pastoralist migration to other ecological zones were made. Each of the aforementioned climatic dimensions is assigned a symbolic meaning and significance communicated in language, ritual, and religion.

## Conclusion and Recommendations

The study concludes that the interaction between the pastoralists and their natural environment is important in sustaining the pastoralist livelihood system. The pastoralist livelihood system is based on livestock, especially cattle. However, increasing changes in the natural circumstances due to the loss of water and forage resources have been a source of a number of problems faced by pastoralists in their interaction with the natural environment.

These changes in the natural circumstances faced by pastoralists are by no means without sociological significance. In the course of interacting with nature, pastoralists ascribe meanings to different characteristics of changing natural situations. Such perceptions are derived from interactions with both nature and their social peers. Of interest to the sociology of migration are the perceptions associated with decisions to migrate in the wake of the increasing changes in their natural and social circumstances that the pastoralists confront.

One conclusion that can be drawn from the study's findings is that before an ultimate decision to migrate is made, pastoralists test various response mechanisms to adopt and adapt to changing circumstances. Changing perceptions among households are an important factor that comes into play before a decision to migrate is made. The adoption of science and innovation was one of the response mechanisms to ward off migration. Others were protecting traditional land-use patterns. However, as perceptions change, particularly through micromigration within the environs, changes to the family structure and to gender relations, and social interactions with peers, a final decision to migrate is made. It is important for the growing body of sociology of migration in Africa to understand the contribution that perceptions and social interaction with peers make to migration.

The definitive conclusion of the study is that pastoralists define their interactions through perceptions developed in the course of interactions with both nature and their social peers. The study concludes that an understanding of perceptions, as brought out in this study, is important for understanding the social basis of migration. A further understanding of perceptions in terms of the subjective views of pastoralist households in the space of migration will help to understand the causes of migration and how they can be managed. It is well-known that different perceptions among pastoralists, agro-pastoralists, and agriculturalists have been the source of a number of social problems, including inter-ethnic conflict. The study's findings show the centrality of understanding pastoralists' expectations compared to the different livelihood groups they encounter. Moreover, the study concludes that individual perceptions of the various stimuli encountered in the course of their interaction with their natural and social circumstances are key to understanding migration resulting from changing social circumstances among pastoralist households.

The methods that were adopted had three notable limitations. First, the majority of the pastoralist households surveyed were confined to the originating areas of pastoralist migration. With the exception of Kiteto, which was an intermediate migration zone, and Mvomero and Iringa (Rural), which were pastoralist migration destination zones, the other survey districts were in long-term pastoralist settlement areas. Likewise, two of the case-study villages for the KIIs and participatory observation and descriptive studies, Mkurlu and Uwiro, were in the pastoralist migration originating area in the north. Msowero village, a pastoralist migration destination area, was in central Tanzania. The implication of this was that the study obtained less information on the indigenous knowledge (IK) lost through migration. The study calls for future research to focus on case studies of the IK actually lost through particular migration paths taken by pastoralists migrating to southern parts of Tanzania. The study takes note of this limitation and recommends that future research on pastoralist migration in Tanzania should focus on areas such as Mbeya, Lindi, Coast, and Tanga.

The second limitation was that the study interviewed pastoralist organizations that are participating in the national Pastoralist Programme (PP). This may have contributed to sampling bias in favor of larger organizations compared to smaller community-based organizations directly operated by the pastoralists themselves^7^.

Further research is needed on the sustainability of pastoralism as a livelihood system in the foreseeable future. Further studies will need to focus on whether an ecological loss is imminent in the context of climate change and extreme weather conditions. There should be a particular focus on whether changes in perceptions regarding the adoption of new technology and innovations to improve interaction with their natural circumstances can delay pastoralists' decisions to migrate. Further research can take stock of and identify the complement of options available to pastoralists to adapt to increasing climate change, including transforming their livelihood systems, perceptions, mindset, and cosmology in light of the changes. This will contribute to our common understanding of the nature of intra-African migration in light of changing weather patterns and ecological loss. This includes an identification of emerging disease patterns and epidemics that were previously under control but over which control now appears to have been lost.

The study calls for future research to document IK lost through pastoralists' migration to other ecological zones in the southern parts of Tanzania. Most studies have focused on pastoralist emigration areas and less on the areas where they settle. As a result, little has been documented concerning their experiences and changing perceptions as they interact with IK loss along their migration path. Research is also needed on the complement of alternative land-use and settlement patterns that can be adopted to sustain and assert ecological control in changing weather conditions.

## Data Availability Statement

The datasets generated for this study are available on request to the corresponding author.

## Ethics Statement

The studies involving human participants were reviewed and approved by Open University of Tanzania Research and Publication Committee. The patients/participants provided their written informed consent to participate in this study.

## Author's Note

This is my original work conducted from original research. This work was previously submitted in the Frontiers in Sociology, section Medical Sociology but was not admitted in the initial review. I was advised to resubmit. I have now worked on the comments and decided to resubmit in this sociology and migration section as I find this to be more relevant to the Article.

## Author Contributions

The author confirms being the sole contributor of this work and has approved it for publication.

## Conflict of Interest

The author declares that the research was conducted in the absence of any commercial or financial relationships that could be construed as a potential conflict of interest.

## Footnotes

^1^Among these, 49.63% said they could not find any solutions to their climate change problems, and 23.62% said they did not know; 0.37% of those surveyed did not respond.

^2^The distribution of respondents to the semistructured quantitative household questionnaires was 23.06% female and 76.94% male. The higher proportion of males was attributed to a higher proportion of heads of pastoralist households being men. 89.67% were married. The highest level of education completed by 43.55% of respondents was primary school. 39.85% did not have any form of formal education. 11.07% had received secondary education. 60.89% had a household size of 6–15 members. Large households with 26–50 members and more than 50 members accounted for 4.61 and 2.4%, respectively. 18.08% of the households surveyed had five members or fewer.

^3^The key informant interviews and observation studies were conducted in three case-study villages in Mkurlu and Uwiro in Arusha Rural District and Msowero in Kilosa District. Arusha Rural District is a predominantly pastoralist push migration district, and Kilosa is a destination district.

^4^The structured quantitative household questionnaires were conducted in 544 households in 7 districts. The sampled districts, their geoclimatic zones, and sample size in brackets were Longido, Hanang, and Kiteto districts in the Northern Zone (184 households), Mvomero district in the Central Zone (120 households), Iringa Rural district in the Southern Highland Zone (86 households), and Bunda and Geita districts in the Lake Zone (154 households). The agro-ecological zones were chosen through a purposive judgmental sampling procedure targeting districts with the highest proportion of households that reported livestock keeping as their main economic activity in the National Sample Census of Agriculture 2002/2003: Livestock Sector Report (National Bureau of Statistics, 2014). The second criterion was to distribute the sample across the four main agro-pastoralist geoclimatic zones using the probability proportion to size (PPS) method. Within the districts, sampling of pastoralist households was done through simple random sampling. The FDGs involved women and girls, youth, and mixed community groups, while the KIIs involved elderly people; community, clan, and household heads; and community leaders. The distribution of FGDs was 5 female-only FGDs, 5 youth, 5 male, and 2 mixed groups.

^5^Cassette tape recorders were used during the qualitative in-depth FGDs, and in all instances, the group participants were clearly informed of their intended use, and the eventual archival and disposal of the tape recording and protection of the identity and confidentiality of respondents were explained and followed. All study respondents were anonymous, thus protecting the individual identities of the respondents. None of the respondents was a minor below 18 years of age.

^6^The passages quoted in this section are excerpts from the interviews with pastoralist community members conducted for this study.

^7^The following five measures were taken to minimize bias. The first was to increase internal validity and ensure that the sample survey data were representative. The first step taken was the adoption of the PPS method, which ensured that the total targeted sample for the semistructured quantitative questionnaire of 500 pastoralists households was proportionally distributed among the seven study districts according to the proportion of number of households with livestock keeping as main economic activity as reported by the 2003 Livestock Census. Second, the study minimized bias attributed to the construction of the study questions by pretesting both the semistructured and unstructured questionnaires through peer interviews among field enumerators. Third, the study minimized sampling bias through systematic selection of household respondents through simple random selection. This was arrived at by first obtaining a list of all households in the village and then choosing every “nth” household based on the number of households required to be interviewed. This measure ensured that each household had an equal chance of being included in the study sample. Fourth, the study conducted a post-coding exercise to eliminate recording errors. Fifth and finally, the enumerators were paired up to reduce errors during data entry.
